# Impact of erythrocytes on bacterial growth and antimicrobial activity of selected antibiotics

**DOI:** 10.1007/s10096-018-03452-4

**Published:** 2019-01-28

**Authors:** Alina Karoline Nussbaumer-Pröll, Sophie Knotzer, Sabine Eberl, Birgit Reiter, Thomas Stimpfl, Walter Jäger, Stefan Poschner, Markus Zeitlinger

**Affiliations:** 1 Department of Clinical Pharmacology, Medical University of Vienna, Vienna, Austria; 2 Clinical Department of Medical and Chemical Laboratory Diagnostics, Medical University of Vienna, Vienna, Austria; 3 Divison of Clinical Pharmacy and Diagnostics, University of Vienna, Vienna, Austria

**Keywords:** Erythrocytes, MHB, In vitro, Pharmacodynamics (PD), MIC, TKC, *Escherichia coli* ATCC25922, *Staphylococcus aureus* ATCC29213, *Pseudomonas aeruginosa* ATCC27853

## Abstract

It has been shown that protein binding, temperature, and pH influence in vitro pharmacodynamic (PD) models. The fact that corpuscular blood compounds might also have an important impact is something which has, until now, often been neglected. We investigated if the addition of human erythrocytes to standard growth media (Mueller Hinton Broth, MHBII) has an influence on bacterial growth behavior and on antibiotic efficacy. We did this by using bacterial growth assays and time kill curves (TKC) of selected strains (*Escherichia coli* ATCC25922, *Staphylococcus aureus* ATCC29213, and *Pseudomonas aeruginosa* ATCC27853) over 24 h. The final concentration of erythrocytes was set to match the physiological concentrations in the blood of a healthy human, i.e., 3 × 10^6 cells/μl in MHBII. Meropenem, ciprofloxacin, and tigecycline were tested with concentrations several-fold above and below the minimal inhibitory concentration (MIC). Moreover, HPLC analysis of antibiotic stability and distribution in erythrocytes was performed. Meropenem, ciprofloxacin, and tigecycline showed the greatest decline in activity against *E. coli* when erythrocytes were present. A mean difference in log10 bacterial killing between pure MHBII and 50%-Ery of 3.83, 1.33, and 2.42 was found for ciprofloxacin, meropenem, and tigecycline, respectively. In the case of ciprofloxacin, HPLC analysis revealed that less extracellular antibiotic is available in the presence of erythrocytes. We have demonstrated that erythrocytes do influence antimicrobial activity and that this might have an impact on the extrapolation of in vitro activity testing to in vivo efficacy in patients.

## Introduction

In both antimicrobial drug development and post-marketing dose optimization, in-vitro pharmacodynamic (PD) models are used to predict efficacy. Time-kill curves (TKC) and minimal inhibitory concentrations (MIC) are frequent methods used for in vitro susceptibility testing.

Attempts are often made to apply in vitro experiments to in vivo situations, which is challenging because bacterial growth media often lack important host factors, such as plasma proteins, different pH settings, or host cells.

Protein binding (PB) is considered a key characteristic of antibiotic activity; therefore, the significance of PB is discussed in various publications. They highlight the difficulty of the standardization of PB measurement and quantification, the impact of supplements to mimic in vivo serum protein concentrations, and how other factors such as pH, temperature, and electrolytes may influence the PB and therefore antibiotic activity [1–3].

Moreover, pH is a well-investigated factor as acidification of human urine is a widely recommended practice for prophylaxis and treatment of urinary tract infections. The in vivo pH of human urine varies from 5 to 7 and studies show that there is a correlation between acidification and decreased antibiotic activity [4, 5].

Furthermore, the presence of Mg^2+^ and Ca^2+^ can also affect antibiotic activity and cation-dependent inhibition has already been reported for polymyxins. The concentration recommended by the Clinical and Laboratory Standard Institute (CLSI) for supplementation of Mueller Hinton Broth (MHB) with these ions is markedly lower than in vivo interstitial space fluid (ISF) and therefore, experiments in standard growth media might overestimate the antibiotic activity in vivo [6].

In addition to modification of individual factors, other studies aiming to reflect physiological conditions directly employ samples of bodily fluids. Studies with cerebrospinal fluid (CSF) show that clinical efficacy of antibiotics is often less than that noted in standard growth media [7].

In vitro studies demonstrate that for *Staphylococcus aureus* (*S. aureus*), higher concentrations of fosfomycin and linezolid are needed in CSF compared to MHB [8, 9]. Similar impaired antibiotic activity is found in human bile when compared with standard growth media [10].

However, even though these various host factors have been investigated, the impact of corpuscular components like red blood cells is poorly understood and therefore often neglected.

We set out to investigate if the addition of human erythrocytes to standard growth media has the potential to influence bacterial growth behavior as well as susceptibility towards antibiotics. Meropenem, ciprofloxacin, and tigecycline were chosen as representatives of broad-spectrum antibiotics with different chemical qualities.

## Methods

### Bacterial strains

Bacterial growth assays and TKCs were performed with selected control strains *S. aureus* (ATCC® 29213™), *Escherichia coli* (*E. coli*) (ATCC® 25922™), and *Pseudomonas aeruginosa* (*P. aeruginosa*) (ATCC® 27853™) obtained from the American Type Culture Collection (ATCC®) (American Type Culture Collection, Manassas, VA, USA)

### Antibiotics

For susceptibility testing and pharmacodynamic experiments, the antibiotics meropenem (trihydrate powder, Sigma-Aldrich, Steinheim, Germany), ciprofloxacin (parenteral infusion solution, Ciprofloxacin Kabi®, Fresenius Kabi Austria GmbH, Graz, Austria), and tigecycline (hydrate, Tygacil, Sigma-Aldrich, Steinheim, Germany) were used

### Growth media

Cation adjusted Mueller Hinton II Broth (MHBII) (Sigma-Aldrich, Steinheim, Germany) containing 17.5 g/l casein acid hydrolysate, 2 g/l beef extract, and 1.5 g/l starch adjusted to a final pH of 7.4 ± 0.2 was used as liquid medium for all organisms tested with or without erythrocytes.

#### Red blood cells

Leukocyte-depleted, sterilized erythrocyte concentrates for transfusion with a hematocrit of ~ 60% stabilized with 70 ml citrate-phosphate-dextrose buffer per 100 ml concentrate (final pH 7.1–7.2) were obtained from the Department of Blood Serology and Transfusion Medicine at the General Hospital of Vienna (General Hospital, Vienna, Austria). The final concentration of the erythrocytes in our experiments was set to match physiological concentrations in the blood of healthy humans, i.e., an erythrocyte concentration of 3 × 10^6 cells/μl in cation adjusted MHBII.

#### Bacterial growth with native and lysed erythrocytes

The impact of erythrocytes on growth of bacterial strains was tested with growth assays performed over 24 h in a water bath at 37 °C under aerobic conditions with 10%, 30%, and 50% erythrocyte-MHBII mixtures in comparison to lysed erythrocyte-MHBII medium mixtures in 14-ml falcon tubes. Red blood cells were added prior to inoculation with the bacteria. The bacterial suspension was adjusted to 1.5 × 10^8^ cells/ml in NaCl, corresponding to a McFarland standard of 0.5, and was added to the test tubes at a final concentration of 1.5 × 10^6^.

Erythrocyte lysis was done by freeze thawing of the cells. Freshly prepared erythrocyte aliquots were stored at − 80 °C for a minimum of 24 h following thawing of cells at 37 °C in a shaking water bath. Cell lysis was confirmed visually under the light microscope (Carl Zeiss Microscopy GmbH, Munich, Germany) by taking a volume of 20 μl and spreading the sample homogenously on a glass slide (Karl Hecht, Sondheim, Germany).

#### Optical and quantitative analysis of intact native erythrocytes

To check the integrity of native erythrocytes within TKC experiments, control samples of 6 ml 50% erythrocyte-MHBII mixtures and samples with additional bacterial suspension of *E. coli*, *S. aureus*, and *P. aeruginosa* at a final concentration of 1.5 × 10^6^ bacterial cells were generated in triplicates and incubated at 37 °C for 24 h. Erythrocyte-MHBII mixtures were checked visually under the microscope before and after incubation as mentioned above. Additionally, a quantitative analysis of intact erythrocytes was done with a hematology analyzer XE-5000 (Sysmex, Austria GmbH), before and after incubation.

### MIC

Determination of the MIC for all ATCC® strains was done according to the performance standards for antimicrobial susceptibility testing of the Clinical and Laboratory Standards Institute (CLSI) (National Committee for Clinical Laboratory Standards) in pure MHBII.

### TKC

All TKC analyses were performed over 24 h in a water bath at 37 °C under aerobic conditions with 6 ml of 50% erythrocyte-MHBII mixtures in comparison to pure MHBII medium in 14-ml falcon tubes. Red blood cells were added prior to inoculation with the bacteria. The bacterial suspension was adjusted to 1.5 × 10^8^ cells/ml in NaCl, corresponding to a McFarland standard of 0.5, and was added to the test tubes at a final concentration of 1.5 × 10^6^. Concentrations were simulated several-fold above and below the MIC, resulting in five different antibiotic concentrations. Experiments included triplicates of all concentrations in adapted and pure MHBII as well as duplicates for the growth controls in MHBII with and without erythrocytes. Samples were taken at time point 0 (before the addition of antibiotics) and then at 3, 7, and 24 h. Subsequently, seven serial dilution steps were carried out in 96-well microtiter plates filled with 0.9% NaCl. These were then dropped onto Columbia blood agar plates and incubated at 37 °C under aerobic conditions for 24 h. After incubation, the colony forming units (CFUs) were counted and the CFU/ml was calculated by taking the dilution steps into consideration. This was done using the following equation: number of CFU multiplied by 5 × 10^x^, where *x* represents the dilution number.

### Evaluation of stability and distribution of antibiotics

To evaluate whether a potential impact on antibiotic activity is caused by the effects on the stability of the antibiotic or because of distribution, e.g., diffusion of antibiotic into erythrocytes, HPLC analysis was done to determine the quantity of free antibiotic in two different settings. Within adapted 50% Ery-MHBII medium and reference medium MHBII, samples were generated with a ciprofloxacin and meropenem setup at a final concentration of 0.1 μg/ml and a further experimental setup with tigecycline 1 μg/ml.

### Chemical analysis

#### Preparation erythrocyte samples

As has been mentioned above, 6 ml 50% Ery-MHBII mixtures were prepared in 14-ml falcon tubes, in triplicate, for time points 0, 3, 7, and 24 h. Both ciprofloxacin and meropenem were added to each sample resulting in a final concentration of 0.1 μg/ml in one tube. All samples were incubated at 37 °C in a water bath until the relevant time point (this does not apply to the sample taken at time point 0). Samples were vortexed and centrifuged at 37 °C with 250× g for 10 min. For HPLC analysis, 1000 μl of the supernatant were pipetted in 1.8-ml Eppendorf tubes, blast frozen at − 21 °C, and then stored at − 80 °C until analysis. The remaining blood clot was also frozen in the 14-ml falcon tubes and ciprofloxacin and meropenem concentrations were semi-quantitatively analyzed by HPLC. This procedure was performed for all time points. Samples with tigecycline were prepared in the same way as the ciprofloxacin-meropenem samples with a final concentration of 1 μg/ml, but without a semi-quantitative blood clot analysis. This decision was made since the pre-analytical methods would have impacted the stability of tigecycline.

#### Preparation MHBII samples

Additionally, samples with 6 ml of MHBII using the same final concentrations of ciprofloxacin-meropenem and tigecycline were generated in 14-ml falcon tubes alongside the abovementioned experiments and served as a reference.

#### HPLC analysis of ciprofloxacin, meropenem, and tigecycline

Ciprofloxacin (No. 17850) and meropenem trihydrate (No. 32460) were obtained from Sigma, Saint Louis, MO, USA, ciprofloxacin-d8 (C48250) and meropenem-d6 (M225617) from Toronto Research Chemicals, North York, Canada. Tigecycline hydrate, tygacil, was obtained from Sigma-Aldrich, Steinheim, Germany.

#### LC-MS/MS conditions and sample preparation

All ciprofloxacin and meropenem samples, supernatant, blood clots, and MHBII reference were thawed at room temperature. Seventy microliters of MHBII and 10 μl internal standard solution (10 μg meropenem-d6 and 10 μg ciprofloxacin-d8/ml) were transferred to the sample tubes, precipitated with 150 μl MeOH, vortexed for approx. 10 s and then centrifuged for 5 min at 14.000×*g*. One hundred fifty microliters of the supernatant were transferred into an autosampler vial, diluted with 450 μl water, and vortexed. One liter was injected into the LC-system.

Ciprofloxacin and meropenem samples were analyzed using liquid chromatography-tandem mass spectrometry with a 5500 Qtrap system (Sciex, Framingham, MA, USA) equipped with a TurboIon Source for electrospray ionization. The chromatographic system consisted of a Symbiosis ALIAS chromatographic system (Spark Holland B.V., Emmen, Netherlands).

High-performance liquid chromatography (HPLC) with a Kinetex F5 (2.6 μm, 100 Å, 100 × 2.1 mm, Phenomenex, Torrance, CA, USA) was used. Mobile phase A consisted of 2 mmol ammonium acetate in 0.1% aqueous formic acid (*v*/*v*), mobile phase B of 2 mmol ammonium acetate in 0.1% methanolic formic acid at a flow rate of 0.30 ml/min and a gradient elution program as follows: 96% A with a linear decrease to 45% A over 1.45 min and a return to 96% A at 3 min (hold 1 min). The equilibration time was 3 min.

The mass spectrometer was operated in the positive mode and quantification was performed by multiple reaction monitoring (MRM). The following mass transitions were used for meropenem *m/z* 384.1 ➔ 340.0, for d6-meropenem (internal standard) *m/z* 390.0 ➔ 346.1, for ciprofloxacin *m/z* 332.0 ➔ 230.9, and for ciprofloxacin-d8 (internal standard) *m/z* 340.3 ➔ 296.2, respectively.

For the determination of ciprofloxacin and meropenem in the erythrocyte experiment, MHBII was used for establishing calibrators and quality controls in the range of 0.05 to 1 μg/ml.

The concentration of tigecycline samples in MHBII and 50% Ery-MHBII was determined by HPLC according to literature [10]. Briefly, frozen samples were thawed at room temperature. After the addition of 600 μl of ice-cold methanol to 200 μl of MHB medium, the samples were centrifuged (13,000*g* for 5 min) and 100 μl of the clear supernatant injected onto the HPLC column. The determination of tigecycline was performed using a Dionex “UltiMate 3000” system (Dionex Corp., Sunnyvale, CA) with UV detection at 348 nm. Chromatographic separation was carried out at 45 °C on a Hypersil BDS-C18 column (5 μm, 250 × 4.6 mm I.D., Thermo Fisher Scientific, Inc., Waltham, MA), preceded by a Hypersil BDS-C18 precolumn (5 μm, 10 × 4.6 mm I.D.) at a flow rate of 1.0 ml/min. The mobile phase A consisted of potassium phosphate (50 mM, pH 3.0 with phosphoric acid) and heptanesulfonic acid (5 mM) and the mobile phase B consisted of methanol. The gradient ranged from 0% B (0 min) to 60% B at 15 min was kept constant at 60% until 20 min and then decreased linearly to 0% B by the 22-min time point. The columns could re-equilibrate for 8 min between runs. Linear calibration curves were performed from the peak areas of tigecycline to the external standard by spiking drug-free MHB medium with standard solutions of tigecycline to obtain a concentration range of 0.02 to 2 μg/ml (average correlation coefficients > 0.999). For this method, the limit of quantification for tigecycline was determined to be 20 ng/ml in MHB medium (coefficients of accuracy and precision were < 9%).

## Results

### Growth controls

**Fig. 1:**
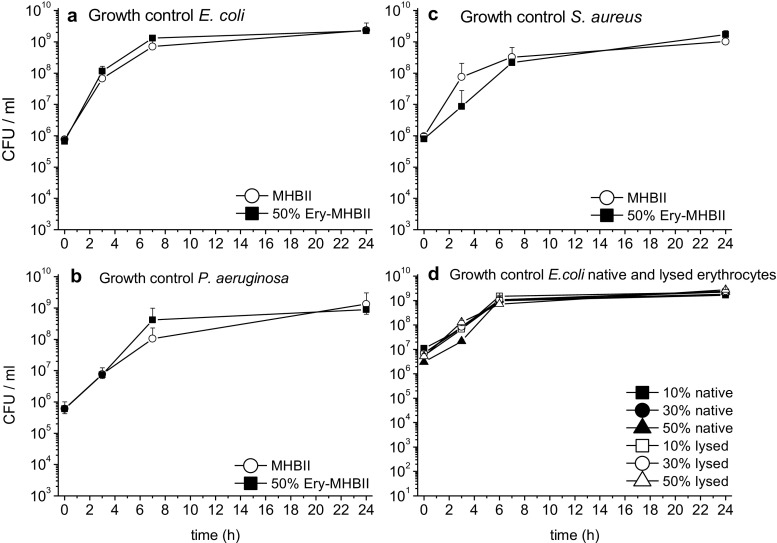
Mean CFU/ml with standard deviation are shown for *E. coli* (**a**), *P. aeruginosa* (**b**), and *S. aureus* (**c**) at time points 0, 3, 7, and 24 h. The white circle represents reference media MHBII and the black squares represent the adapted media with 50% Ery. Furthermore, growth controls for *E. coli* with native (black symbols) and lysed (white symbols) erythrocytes with 10%, 30%, and 50% red blood cells are shown (**d**)

In Fig. 1, the growth curves of *E. coli* (a), *S. aureus* (b), and *P. aeruginosa* (c) over 24 h are shown for pure MHBII and for the adapted 50% Ery-MHBII medium.

No significant impact was seen for the growth of *E. coli*. *S. aureus* showed slightly impaired growth for 50% Ery after 3 h compared to pure MHBII which was no longer present at 7 h. *P. aeruginosa* showed a slightly better growth with the 50% Ery-MHBII mixture regarding the 7-h time point. Furthermore, growth controls for *E. coli* with native and lysed erythrocytes with 10%, 30%, and 50% red blood cells are shown (d). No difference in growth could be observed between lysed and native erythrocytes for gram + and gram − bacteria.

**Fig. 2:**
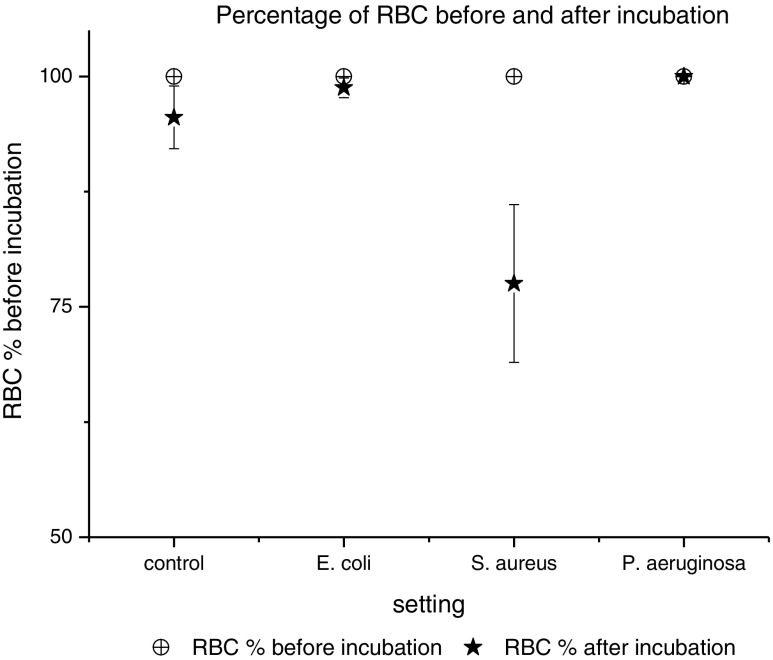
The percentage of red blood cells (RBC) before (white symbols) and after incubation (black symbols) at 37 °C over 24 h, with and without bacterial strains is shown

In Fig. 2, the integrity of native erythrocytes before and after 24 h at 37 °C is compared in percentage. No significant difference in the erythrocyte count after incubation with *E. coli* and *P. aeruginosa* was seen. Contrary, with *S. aureus* up to 25%, less intact erythrocytes were found after incubation.

### Bacterial killing

**Fig. 3:**
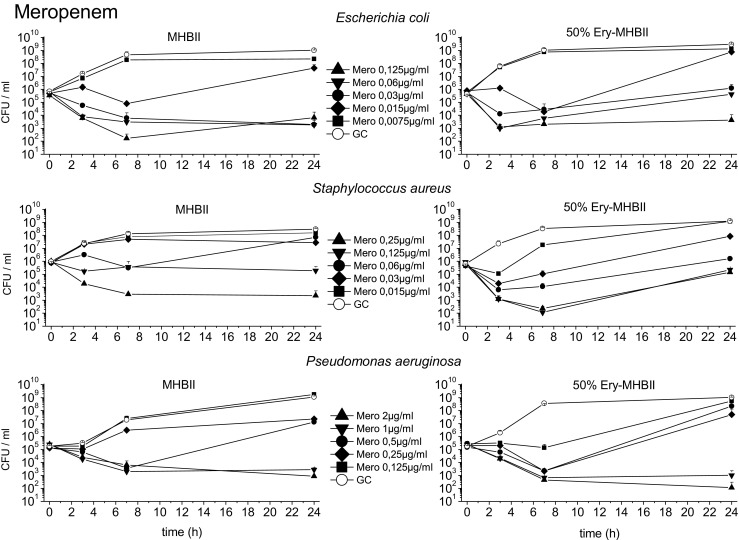
The mean data with standard deviation of the TKCs of *E. coli*, *S. aureus*, and *P. aeruginosa* tested with meropenem in reference media MHBII and adapted media 50% Ery-MHBII over 24 h are shown

**Fig. 4:**
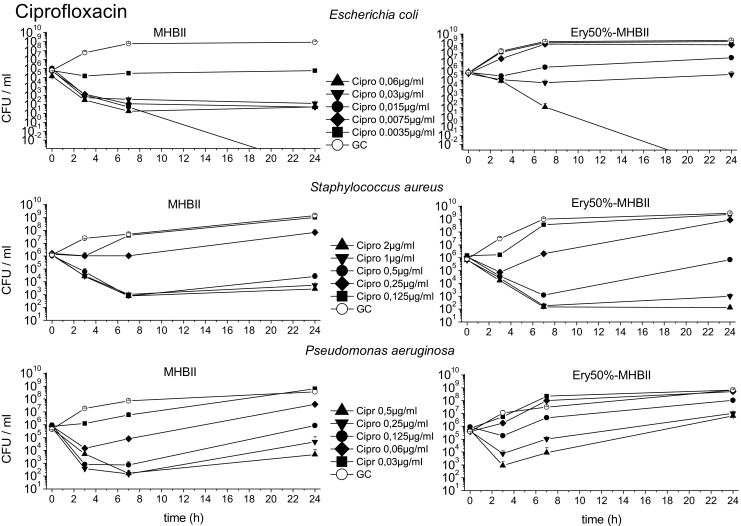
The mean data with standard deviation of the TKCs of *E. coli*, *S. aureus*, and *P. aeruginosa* tested with ciprofloxacin in reference media MHBII and adapted media 50% Ery-MHBII over 24 h are shown

**Fig. 5:**
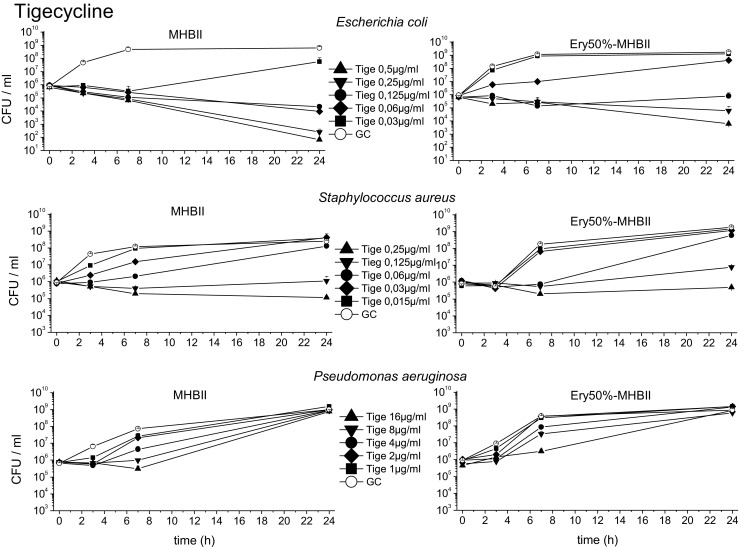
The mean data with standard deviation of the TKCs of *E. coli*, *S. aureus*, and *P. aeruginosa* tested with tigecycline in reference media MHBII and adapted media 50% Ery-MHBII over 24 h are shown

TKC with meropenem, ciprofloxacin, and tigecycline are shown in Figs. 3, 4, and 5. Standard MHBII reference media and adapted 50% Ery-MHBII media are compared for *E. coli*, *S. aureus*, and *P. aeruginosa*.

The average log10 difference before (0 h) and after (24 h) antibiotic exposure is shown in Table 1 for all tested bacterial strains and antibiotics in reference media MHBII and in 50% Ery-MHBII media. In addition, the delta in log10 bacterial killing after 24 h ($\frac{\mathrm{CFU}\ 24\mathrm{h}}{\mathrm{CFU}\ 0\mathrm{h}}$ 50% Ery − $\frac{\mathrm{CFU}\ 24\mathrm{h}}{\mathrm{CFU}\ 0\mathrm{h}}$ MHBII) between pure MHBII and 50% Ery are depicted.

**Table 1:** It presents the impact of erythrocytes on the bacterial killing tested with the different antibiotics and bacterial strains. The first two columns show the average difference in log10 killing before (0 h) and after (24 h) antibiotic administration in pure MHBII and in adapted media. In the right column, the 50% Ery-MHBII media is compared with pure MHBII as delta of average log10 CFU/ml differences for all investigated concentrations

	*Escherichia coli*		
	MHB II $\overline{x}\ \mathit{\log}10\ \left(24h/0h\right)$	50% Ery-MHBII $\overline{x}\ \mathit{\log}10\ \left(24h/0h\right)$	∆
Meropenem	− 0.45	0.89	1.33
Ciprofloxacin	− 4.40	− 0.58	3.83
Tigecycline	− 1.85	0.58	2.42
	*Staphylococcus aureus*		
	MHB II $\overline{x}\ \log 10\ \left(24\mathrm{h}/0\mathrm{h}\right)$	50% Ery-MHBII $\overline{x}\ \log 10\ \left(24\mathrm{h}/0\mathrm{h}\right)$	∆
Meropenem	0.51	0.97	0.46
Ciprofloxacin	− 1.25	− 0.98	0.27
Tigecycline	1.33	1.94	0.61
	*Pseudomonas aeruginosa*		
	MHB II $\overline{x}\ \log 10\ \left(24\mathrm{h}/0\mathrm{h}\right)$	50% Ery-MHBII $\overline{x}\ \log 10\ \left(24\mathrm{h}/0\mathrm{h}\right)$	∆
Meropenem	0.76	0.63	− 0.14
Ciprofloxacin	0.29	2.12	1.83
Tigecycline	3.12	3.16	0.03

Overall, the TKCs showed a decrease in antibiotic activity for most of the bacterial strains when erythrocytes are present.

The strongest decrease in antibiotic activity was seen for *E. coli* and ciprofloxacin with a mean delta log10 value of 3.83. Meropenem and tigecycline showed a decrease with a mean delta of 1.33 and 2.42, respectively. A slightly attenuated but nonetheless negative effect on antibiotic activity was seen for *S. aureus* with meropenem, ciprofloxacin, and tigecycline with a mean delta of 0.46, 0.27, and 0.61, respectively.

Although a decrease in activity was also seen for *P. aeruginosa* when tested with ciprofloxacin (mean delta of 1.83), no effect could be seen for meropenem and tigecycline with a value of − 0.14 (i.e., 50% Ery minimally promote antibiotic activity) and 0.03, respectively.

**Fig. 6:**
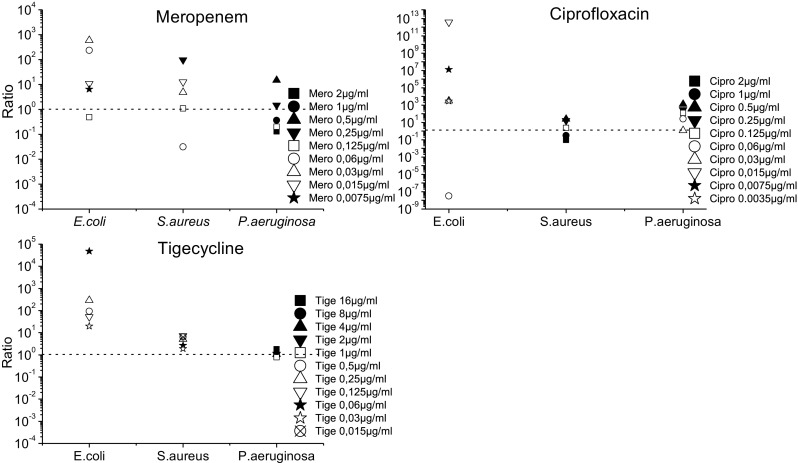
The TKC results are presented as ratios (($\frac{\mathrm{CFU}\ 24\mathrm{h}}{\mathrm{CFU}\ 0\mathrm{h}}$ 50%Ery) ÷ ($\frac{\mathrm{CFU}\ 24\mathrm{h}}{\mathrm{CFU}\ 0\mathrm{h}}$ MHBII)). Ratios above 1 indicate a decrease in antibiotic activity when erythrocytes are present, and ratios below 1 show increased antibiotic activity. When the ratio is around 1, the addition of erythrocytes demonstrates no effect

To depict the impact of addition of erythrocytes at different concentrations in relation to the MIC, the ratios “adapted medium/pure MHB” in log10 change of CFU/ml compared to baseline are plotted in Fig. 6.

The ratios in Fig. 6 indicate a decrease in antibiotic activity in the presence of erythrocytes when they are above 1 and an increase in antibiotic activity when they are below 1. Ratios of around 1 show that the addition of erythrocytes had no effect. *E. coli* shows for most antibiotics and concentrations a ratio above 1, except for the highest tested concentration of meropenem 0.125 μg/ml and ciprofloxacin 0.06 μg/ml. *Staphylococcus aureus* shows ratios mainly above 1 or around 1, except for the concentration 0.06 μg/ml with meropenem which showed marked regrowth in the MHBII medium as seen in Fig. 3. For ciprofloxacin, the two highest concentrations (2 μg/ml and 1 μg/ml) resulted in values below 1. For *P. aeruginosa*, ratios above 1 were found with meropenem and ciprofloxacin; as expected, no effect was seen with tigecycline.

### HPLC analysis

**Fig. 7:**
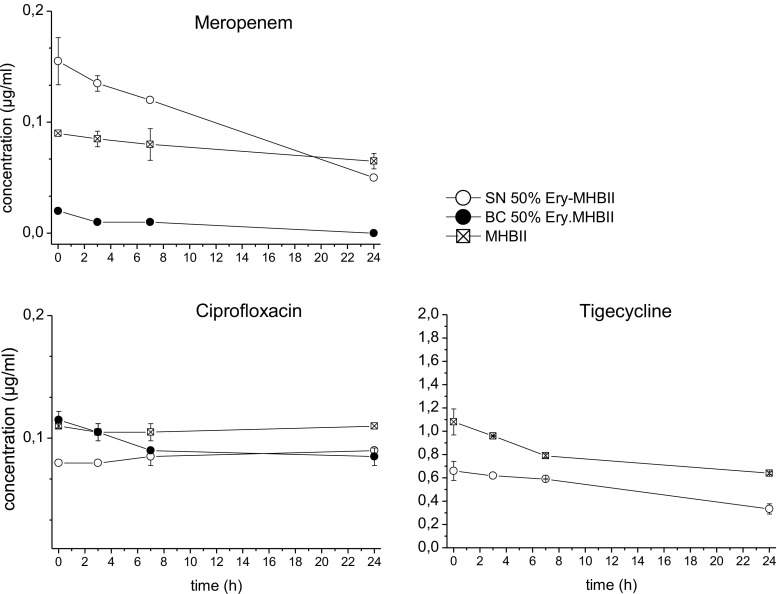
Time-concentrations profiles over 24 h of meropenem, ciprofloxacin and tigecycline with MHBII, supernatant (SN) of 50% Ery-MHBII, and blood clot (BC) of 50% Ery-MHBII

Figure 7 shows time-concentration profiles of meropenem, ciprofloxacin, and tigecycline with the average antibiotic concentration in different compartments from time point 0 to 24 h.

Meropenem with a pre-set concentration of 0.1 μg/ml showed nearly no concentration decrease over time in MHBII. The evaluated concentration of meropenem in the erythrocytes blood clot was nearly zero. In the supernatant, higher concentrations of meropenem were found, but it showed a higher degradation than in pure MHBII. Ciprofloxacin with a pre-set concentration of 0.1 μg/ml was stable in MHBII. Comparing the 50% Ery blood clot and the supernatant, higher concentrations were found in the blood clot.

Tigecycline with a pre-set concentration of 1 μg/ml faced in MHBII and the supernatant a decrease in antibiotic concentration over 24 h; whereas, in the supernatant, lower start and end concentrations were obtained.

## Discussion

Previous studies have shown that under physiological conditions, the presence of erythrocytes promotes bacterial growth in the human body and that bacteria are able to extract hemoglobin and erythrocyte bound iron from red blood cells using various mechanisms and then use it for their metabolism [11, 12]. Nevertheless, until this study was performed, the influence of erythrocytes on antibiotic activity against relevant bacterial strains has been neglected. We demonstrated that the addition of erythrocytes to MHBII had a significant negative impact on antibiotic activity when compared to pure MHBII, thereby confirming that standard bacterial growth media lacks important corpuscular host factors alongside other things and thus might not predict efficacy in vivo. Still, we did not find any impact of erythrocytes on growth of the selected bacterial strains *E. coli*, *S. aureus*, and *P. aeruginosa*, neither in native nor in lysed state. Therefore, we do not expect that our findings will have implications for bacterial blood cultures, at least if they are performed before antibiotic is administrated.

Thus, it can be hypothesized that decreased antimicrobial activity of antibiotics in the presence of erythrocytes may be caused by factors other than promoted growth. Extracellular binding, intracellular accumulation in erythrocytes, or pronounced degradation of the antibiotic in the presence of erythrocytes might be factors to consider, necessitating quantification of the antimicrobials under the investigated conditions as performed in the present study.

Meropenem, ciprofloxacin, and tigecycline showed the highest decline in their activity when erythrocytes were present against *E. coli*. A considerable decrease in activity of all tested antibiotics has been also seen for *S. aureus*; whereas, for *P. aeruginosa*, only ciprofloxacin faced a strong negative impact on its activity in the presence of erythrocytes. These effects could be congruently described by the average delta in log10 bacterial killing between pure MHBII and 50% Ery (Table 1) as well as by the ratios of the TKCs (Fig. 6). As a potential threshold for bactericidal activity in the case of severe infections, a 2 log10 drop of CFU/ml can be assumed [13]. Applying this threshold to achieve a 2 log10 drop with meropenem for *E. coli* in MHBII, 0.03 μg/ml (MIC) was sufficient, whereas, in 50% Ery-MHBII, at least a 4-fold higher dose was needed. *Staphylococcus aureus* demonstrated similar results. Nevertheless, regrowth of all bacterial strains tested with meropenem was observed, especially around the specified MIC concentration. Indeed, antibiotic concentrations around the MIC value may favor selection of resistant strains [14–16]. Only *S. aureus* tested with 0.06 μg/ml meropenem had a stronger regrowth in MHBII than in the presence of red blood cells, which might be driven by a single selected resistant clone.

The HPLC analysis of meropenem showed that initially more antibiotic was found in the supernatant of MHBII with erythrocytes and nearly no meropenem was found in the analyzed blood clot. This supports the fact that meropenem, as a hydrophilic drug, cannot access intracellular space which might favor *S. aureus* as a facultative intracellular pathogen [17]. However, after 24 h, the concentration in 50% Ery-MHBII media was lower than in pure MHBII. The reason for accelerated instability is unclear but might partially explain the decreased antibiotic activity when erythrocytes were present.

The activity of ciprofloxacin also displayed a high negative impact when red blood cells were present. The average delta in log10 bacterial killing between pure MHBII and 50% Ery (Table 1) showed that the bacterial count after 24 h was higher for all tested strains when erythrocytes were present in comparison to MHBII. Moreover, to achieve a 2 log10 killing when red blood cells were present, an 8-fold higher concentration was needed for *E. coli*. An even higher impact of red blood cells was seen for *P. aeruginosa*, where no sufficient killing for a 2 log10 drop was achieved with the tested concentrations within the adapted setup.

The most likely explanation for the decrease in the activity of ciprofloxacin in 50% Ery-MHBII is the excellent penetration properties of ciprofloxacin in tissue and cells, which has been proven by several studies [18, 19]. This might lead to the removal of the available extracellular drug. The HPLC analysis confirmed that ciprofloxacin was stable in MHBII and higher concentrations were found in the blood clot than in the supernatant. One explanation of the limited effect of erythrocytes on *S. aureus* might be that it is a facultative intracellular pathogen [20]. The other more plausible explanation would be the fact that *S. aureus* is able to lyse erythrocytes as seen in Fig. 2. Therefore, more free ciprofloxacin might have been present, compared to the other settings, although this was not specifically investigated.

For tigecycline, the highest decline in activity was found with *E. coli* in the presence of erythrocytes, for which a 2-fold higher concentration in the adapted media was needed to achieve the 2 log10 drop. *S. aureus* was only slightly affected by the red blood cells and *P. aeruginosa* was not affected at all, which might be attributed to the limited activity of tigecycline against *P. aeruginosa* [21]. Moreover, studies prove that oxygenation of tigecycline in MHB might negatively affect its activity [22, 23]. Therefore, we used without exception fresh prepared MHBII to avoid discrepancies between fresh MHB and aged MHB were acceleration of the oxidative inactivation of tigecycline might occur.

HPLC analysis revealed that tigecycline showed a clear degradation over 24 h in MHBII and supernatant. In addition, compared to MHBII, the concentration in supernatant was lower for the whole observation period, which might indicate binding in or at the erythrocytes, resulting in less free antibiotic. Concentration in erythrocytes could not be quantified for technical reasons; however, previous in vitro studies found evidence of significant binding to other proteins such as albumin (71–89%) [21].

In the present study, via binding or penetration into erythrocytes, red blood cells affected the free and therefore, active fraction of ciprofloxacin and tigecycline. This finding should be considered while working with blood samples, because lysis of cells might release bound antibiotic and falsify the free fraction. Therefore, it is important to determine this free fraction before proceeding to PK/PD simulations and to process samples quickly to avoid redistribution ex vivo.

One limitation of this study may be the small number of tested bacterial strains. A broader spectrum of clinical isolates with hemolytic characteristics might have been a useful supplement. Further valuable information would have been the analysis of the tigecycline concentration in the blood clot, which was technically not possible but would have complimented our results, as tigecycline is expected to have a high tissue to plasma ratio as well as good penetration properties in alveolar cells [24]. Future studies should be performed in MHBII and compared to adapted media with serum and erythrocytes to analyze protein binding. Furthermore, the addition of other corpuscular blood components such as thrombocytes could be investigated. Whether our findings would also have impact for solid culture media should be explored in subsequent studies.

To summarize, we have demonstrated that erythrocytes do influence antimicrobial activity which might have an impact on extrapolation of in vitro activity testing to in vivo efficacy in patients and might be a relevant factor for future PK/PD modeling approaches.
